# Evolution and Heredity

**Published:** 1911-02

**Authors:** 


**Evolution and Heredity.** By **David Berry Hart,** M.D., F.R.C.P.E., Lecturer on Midwifery and Diseases of Women, School of the Royal Colleges, Edinburgh. 12 mo, pp. 259. Illustrated. New York: Rebman Co. (Cloth, $2.00)

The questions of evolution and heredity are of enormous significance, theoretical, philosophical, practical. The author has the courage to undertake the discussion of these great questions within the space of a small book; his success is quite remarkable. Aside from the peculiar virtue of being small, this book has distinct value in that it furnishes most intelligent, condensed appreciations of the works of Darwin, Weismann, Gatton, Mendel, and their more prominent critics. This, in our mind, constitutes the real value of the book. Its weakness seems to us to appear as a consequence of the bias with which the author approaches his work. In our judgment this bias is plainly materialistic and unconsciously brings the writer to the tacit conception, that concerning evolution and heredity all that we will ever know, in fact all that is worth knowing, is the mechanism of the same.

Most of those students who maintain that there is in the universe more than the material of which it is constructed, hold that the more intelligent consideration of the facts of evolution, of personality, of heredity, requires constant recognition of at least the possible presence, the possible conspicuous causative operation of the supermaterial.

The bias of the author shows in his habit of treating the hypothetical parts and the imaginary functions of determinants, engams, gametes, mnemism, pangenesis, soma, and zygotes, not as interesting products of the imagination, but as if they were scientific facts, genuine coin from the biometric mind. To our mind the more interesting part of this book, although but slightly related to its subject, are the three chapters upon the habits of the honey bee. The style of the author is delightful. H. R. H.

